# Trabeculotomy opening size and IOP reduction after Trabectome® surgery

**DOI:** 10.1007/s00417-017-3683-0

**Published:** 2017-05-20

**Authors:** Thomas Wecker, Alexandra Anton, Matthias Neuburger, Jens F. Jordan, Christian van Oterendorp

**Affiliations:** 1 Eye Center, Medical Center, Faculty of Medicine, University of Freiburg, Freiburg, Germany; 2 Augenarztpraxis Drs. Neuburger/Burau/Schmidt, Achern, Germany; 3 Berufsausübungsgemeinschaft (GbR) Dr. med. Michael A. Vobig, Prof. Dr. med. Jens Jordan, Frankfurt, Germany; 4 Department of Ophthalmology, University Medical Center Göttingen, Robert-Koch-Str. 40, 37075 Göttingen, Germany

**Keywords:** Glaucoma, Treatment surgery, Angle, Intraocular pressure, Minimally invasive glaucoma surgery (MIGS)

## Abstract

**Background:**

Trabeculotomy with the Trabectome® is an effective surgical procedure to lower intraocular pressure (IOP). However, in some patients it does not lead to a significant IOP reduction despite a gonioscopically well visible opening of Schlemm’s canal. This study investigated whether the size of the trabeculotomy opening and other parameters, including anterior chamber depth (ACD) are related to IOP reduction.

**Methods:**

Retrospective observational case series with 93 eyes of 93 patients who underwent Trabectome surgery. Trabeculotomy opening and ACD were measured with an anterior segment swept source OCT. IOP was taken pre-operatively and at a single follow-up visit [follow-up time 125 ± 66 days (mean ± SD)]. The relationship between IOP reduction and OCT parameters and possible confounding factors was analyzed in a multiple linear regression model.

**Results:**

The trabeculotomy opening size did not correlate with IOP reduction (slope of regression line = 0.0016; 95% confidence interval of slope: −0.025 to 0.028). The same applied for all other parameters tested, including ACD, which showed a tendency towards better IOP reduction with a deeper AC (slope = −1.9; 95% confidence interval: −5.54 to 1.73). Comparison between the 1st and 4th quartile of the trabeculotomy opening showed a significantly higher ACD in the largest trabeculotomy opening quartile (3.32 ± 0.05 mm vs. 3.16 ± 0.04 mm; *p* = 0.031).

**Conclusions:**

The fact that the trabeculotomy opening size did not correlate with IOP reduction points to the poorly understood role of the intrascleral aqueous outflow pathway in glaucomatous IOP elevation. A deeper AC might be a factor promoting a larger trabeculotomy opening.

**Electronic supplementary material:**

The online version of this article (doi:10.1007/s00417-017-3683-0) contains supplementary material, which is available to authorized users.

## Introduction

Various morphological and functional alterations of the trabecular meshwork (TM) are known to increase aqueous outflow resistance in open-angle glaucoma [1, 2]. Thus, partial opening of Schlemm’s canal by ablation of the TM with the Trabectome is supposed to facilitate aqueous drainage to the episcleral veins [3]. Trabectome surgery has been proven a safe and effective procedure to significantly lower intraocular pressure (IOP) in open-angle glaucoma patients [4–6]. During the early post-operative course, the original size of the trabeculotomy opening decreases to a variable degree by re-closure of the TM or formation of anterior synechiae [7] (and personal observation). After 2 years of follow-up, a total of approximately 20 to 40% of all cases (depending on glaucoma type and lens status) fail to develop a significantly lower IOP [8]. Interestingly, some of these failed cases still exhibit a gonioscopically well visible opening of Schlemm’s canal which may indicate an impaired function of the outflow structures distal to Schlemm’s canal, comprising collector channels and the aqueous vein plexus [9]. In fact, various morphological changes of the distal aqueous outflow pathway have been described in glaucoma eyes (summarised in Hann et al., 2011 [10]).

In this retrospective observational case series, we investigated the relationship between the size of the post-operative trabeculotomy opening after Trabectome surgery and the resulting IOP reduction. Given the paradigm of the TM as the main site of aqueous outflow resistance and the observation that Schlemm’s canal is not a continuous tube but compartmentalised, presumably by thin septae, we hypothesized a positive relationship between the trabeculotomy opening and the resulting IOP reduction. This hypothesis was tested using anterior segment spectral domain optical coherence tomography (SD-OCT) and IOP data in a multivariate analysis.

## Materials and methods

### Study design and surgery

Single-center retrospective observational case series. All experiments were performed in accordance with the guidelines of GCP and the Declaration of Helsinki. The study was approved by the University Hospital Freiburg Ethics Committee (permit number 235/10_160678). All patients had given informed consent before inclusion into the study.

Ninety-three eyes of 93 patients with chronic open-angle glaucoma suitable for minimally invasive glaucoma surgery were included. Exclusion criteria were a narrow chamber angle (below Shaffer III) in the nasal quadrant and a history of previous glaucoma surgery including laser trabeculoplasty.

All eyes underwent Trabectome surgery (Trabectome; Neomedix, Tustin, CA, USA) either alone or combined with phacoemulsification and posterior camber lens implantation. All surgery was performed between 2009 and 2015 by three surgeons (AA, MN, JFJ). Trabectome surgery was performed in accordance with the manufacturer’s recommendations. A 1.8-mm clear cornea incision in the temporal quadrant was used to approach the nasal TM. The TM was opened over 4 to 5 clock hours starting with an energy of 0.8 W, increasing in 0.1-W steps until the electrosurgical effect allowed smooth removal of the TM without tearing.

Post-operatively, any topical antiglaucomatous therapy was changed to pilocarpine 2%/timolol 0.5% fixed combination preservative-free eye drops (Fotil sine; Santen GmbH, Munich, Germany) twice daily and topical steroids (dexamethasone 0.1% preservative-free eye drops). In case of timolol intolerance, only pilocarpine and steroids were given. The steroids were tapered within 6 weeks. Topical antiglaucomatous therapy was adjusted during follow-up depending on the post-operative IOP and possible side effects. If IOP readings were higher than 18 mmHg, glaucoma medication was extended.

### Anterior segment OCT and IOP measurements

All follow-up data was obtained at a single visit at approximately 24 weeks (range 6 to 48 weeks) post-operatively (Table 1 and Fig. 2a). If no OCT scan was available at 24 weeks, the follow-up visit with OCT scan closest to the 24-week timepoint was chosen. All OCT scans were taken by a single technician. All measurements of the trabeculotomy opening were performed by a single investigator (CvO) who, at the time of data acquisition and analysis, was blinded for the IOP and other medical details of the patients.

**Table 1:** Data of the study population

Study population (mean ± SD)	
Age	71.1 ± 10.6
Number of eye drops before surgery	2.14 ± 1.09
Number of eye drops at follow-up	1.45 ± 1.21
Follow-up time (days)	125 ± 66
Combined surgery	38 (41%)
Pseudophakic eyes	24 (26%)
Angle opened intraoperatively over XX clock hours	4.5 ± 0.5
Glaucoma subtypes	Number (%)
POAG	58 (63%)
XFG	24 (26%)
Pigment dispersion	3 (3%)
Trauma-associated	2 (2%)
Congenital	1 (1%)
Uveitic	2 (2%)
Low tension	3 (3%)

*POAG* primary open angle glaucoma, *XFG* exfoliation glaucoma

A swept source anterior segment OCT device (Casia SS-1000, Tomey, Erlangen, Germany) was used to obtain 256 radial scans of 16-mm length, each consisting of 512 A-scans. The given number of scans resulted in a scan density of 0.7° per scan.

**Fig. 1:**
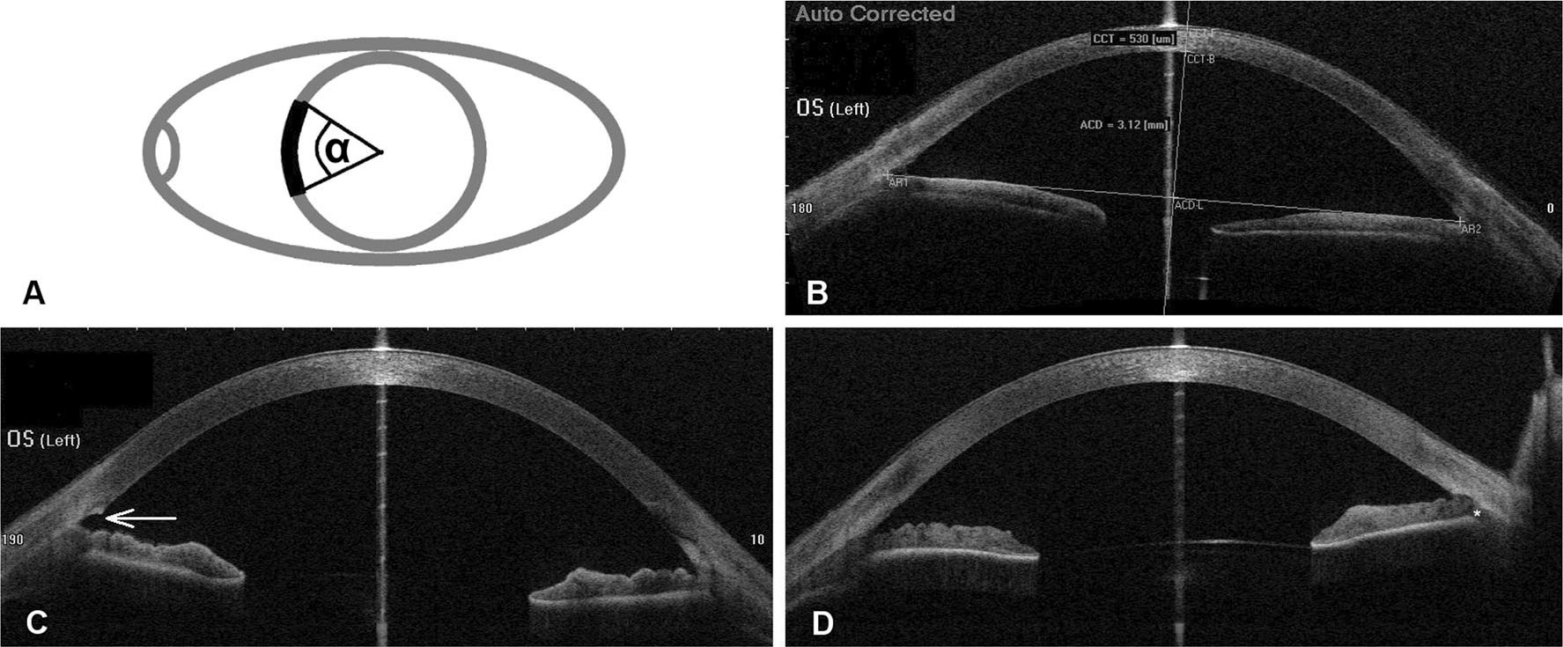
OCT measurements. A) Schematic illustration of the TO opening parameter, defined as the angle α over which the TM appeared open in radial OCT sections. B) Illustration of the ACD measurement. The connecting line between the nasal and the temporal angle recess (AR) served as baseline. The maximum distance perpendicular from the baseline to the posterior surface of the cornea was defined as ACD. C) Representative OCT section showing an open TM (*white arrow*). D) OCT section showing re-closure of Schlemm’s canal by anterior synechia to the TM opening. The scleral spur is located above the white asterisk

The trabeculotomy (TO) opening was defined as the segment of the chamber angle circumference where the TM appeared clearly open (> ¼ of the height of Schlemm’s canal) in the OCT image (illustrated in Fig. 1a, Supplemental video 1). If anterior synechiae, membranes or re-closure of the TM interrupted the segment, only the open sub-segments were counted.

The intra-observer variability of the TO opening measurement was determined by measuring 15 scans twice in random order and in a blinded fashion. Subsequently, the intra-observer intraclass correlation coefficient (ICC) was calculated and the data was plotted in a Bland–Altman chart.

The anterior chamber depth (ACD) was measured in a modified way with the built-in algorithm of the OCT device on a single horizontal OCT section. First, a line between the nasal and the temporal angle recess was drawn (Fig. 1b). Perpendicular to this line the maximum distance to the posterior surface of the cornea was defined as the ACD.

The IOP was measured with Goldmann applanation tonometry (GAT). The baseline IOP was calculated from the mean of three IOP measurements taken the day before surgery. The follow-up IOP was obtained from a single measurement.

The IOP reduction was expressed as either the difference between the pre-operative and the follow-up IOP, named ΔIOP, or as the percentage IOP reduction, calculated as:

$$ {\mathrm{IOP}}_{\mathrm{reduct}}={\left(\left({\mathrm{IOP}}_{\mathrm{follow}-\mathrm{up}}/{\mathrm{IOP}}_{\mathrm{pre}-\mathrm{operative}}\right)-1\right)}^{\ast }\ 100 $$

### Statistical testing

GNU R [11] (version 3.2.2) with additional packages [12, 13] was used for the multiple linear regression model. For additional data analysis of subgroups, either a Student’s *t* test for normally distributed data (tested with D’Agostino–Pearson omnibus K2 test) or a Wilcoxon rank sum test for non-normally distributed data (e.g. percent IOP reduction) was applied, using Prism 6.0 software (GraphPad, La Jolla, CA, USA). Results were either presented as scatter plots with mean and standard error of the mean (SEM) or box plots with whiskers as proposed by Tukey (1.5× interquartile range) and outliers for minimum/maximum values represented by dots [14].

## Results

### Study population and IOP reduction

The relevant data of the study population is summarised in Table 1. An illustration of the parameter TO opening is shown in Fig. 1a. Representative OCT images as used to measure TO opening and ACD are shown in Fig. 1b–d and in supplemental video 1. The mean ± SD TO opening at follow-up was 53 ± 35°, and the mean ACD was 3.24 ± 0.24.

**Fig. 2:**
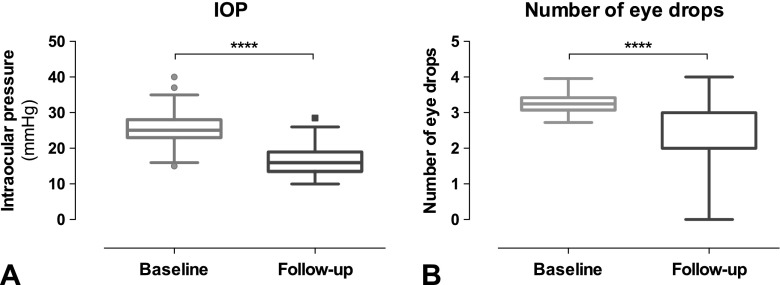
Data of the study cohort. A) Pre- and post-operative IOP (*p* < 0.0001). B) Number of eye drops before surgery and at follow-up (*p* < 0.0001)

After a follow-up time of 125 ± 66 days, Trabectome surgery lead to a significant reduction in IOP (Fig. 2a) with a baseline IOP of 25.2 ± 4.6 mmHg and a mean follow-up IOP of 16.3 ± 4.1 mmHg (*p* < 0.0001; paired *t* test). The number of topical antiglaucomatous medication was significantly reduced from 2.14 ± 1.1 to 1.5 ± 1.2 (Fig. 2b; *p* < 0.0001; Wilcoxon matched-pairs signed rank test).

Surgical success defined as ≥20% IOP reduction and follow-up IOP ≤21 or ≤18 mmHg was reached by 67 eyes (72%) and 60 eyes (65%), respectively.

### Validation of the OCT measurements

To rule out the possibility that an exceptionally high variability of the TO opening measurement blurred the hypothesized correlation with IOP reduction, we determined the intra-observer variability of the TO opening measurement by calculation of the ICC. The intra-observer ICC in a sample of 15 cases measured twice in random order and in a blinded fashion was 0.993, which is considered sufficiently low for not significantly interfering with a potential correlation between TO opening and other parameters. The intra-observer variability data was also plotted in a Bland–Altman chart (supplemental Fig. 1A). The bias and the 95% limits of agreement, calculated from the Bland–Altman chart were 1.7 ± 10.4°, respectively. The slope and 95% confidence interval of the slope of the linear regression analysis were −0.004; −0.05 to 0.04; R^2^ = 0.003. As only one person performed the OCT measurements, no inter-observer variability was calculated.

Thirty-eight eyes (41%) in our study underwent combined Trabectome and cataract surgery. Thus, the lens removal itself might have introduced a systematic error to the ACD measurement. We tested for a systematic change in ACD through cataract surgery with a prospective co-study, using a separate group of 10 eyes of 10 patients, which had all received cataract surgery without any additional procedure. The mean difference (bias) between pre- and post-operative ACD measurements and the 95% limit of agreement was 0.013 ± 0.177 mm (suppl. Fig. 1B). The slope of a linear regression line was −0.057 (95% confidence interval of slope: −0.50 to 0.39; R^2^ = 0.01). Given this data, we assume no significant influence of cataract surgery on our ACD measurement. To further rule out an influence of cataract surgery on our results, a subgroup consisting of all trabectome-only cases (no combined surgery) was separately analyzed (see below).

### Relationship between trabeculotomy opening size, anterior chamber geometry and IOP reduction

**Fig. 3:**
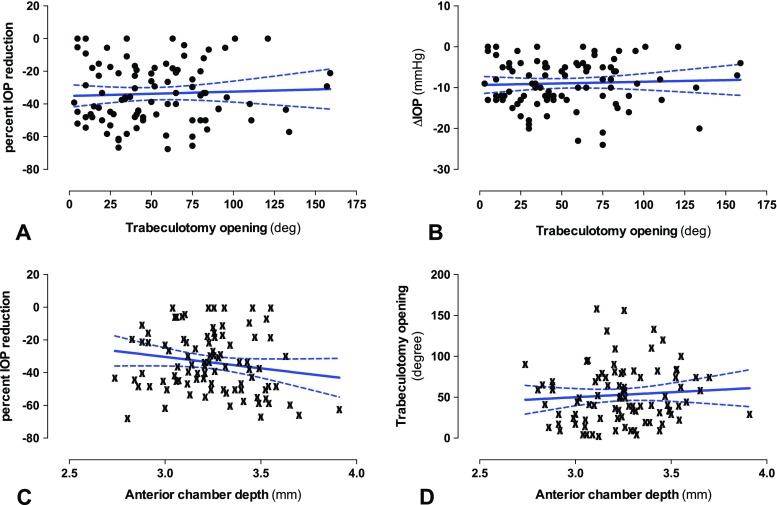
Scatterplots and linear regression analysis for TO opening (*top row*), ACD (*bottom row*) and the IOP reduction. A and B) The TO opening was plotted against the percent IOP reduction (A) or ΔIOP (B). For both cases, the linear regression showed a slope close to zero. C and D) ACD plotted against the percent IOP reduction (C) or the TO opening (D). For higher ACD values, the linear regression line showed a tendency towards better IOP reduction (C) or larger TO opening (D). However, the 95% confidence interval included zero in both cases, which indicates the absence of a linear relationship between the variables

To test whether a larger TO opening correlated with a higher percent IOP reduction, both parameters were plotted in a scatterplot (Fig. 3a) and linear regression analysis was performed. The slope of the regression line was close to zero (slope = 0.03; 95% confidence interval: −0.08 to 0,14; R^2^ = 0.0025), indicating that no relationship between TO opening and IOP reduction existed. The analysis was repeated for ΔIOP (Fig. 3b), producing a similar result (slope = 0.01; 95%CI: -0.02 to 0.04; R^2^ = 0.0024). The same applied for the IOP at follow-up vs. TO opening (data not shown).

A deep AC might have been connected with a greater chance for the trabeculotomy to remain open post-operatively and, thus, might have led to better IOP reduction. We, therefore, plotted the ACD against both parameters (Fig. 3c and d). The linear regression lines showed a tendency towards larger TO opening and better IOP reduction for deeper ACs. However, for both cases, the 95% confidence interval of the slope included the value zero, suggesting that no significant correlation existed.

For a more comprehensive analysis of the influence of TO opening and ACD on the IOP reduction, a multiple linear regression model was set up, which included, besides TO opening and ACD, the following covariates: the change in number of eye drops, lens status (phakic, pseudophakic, combined surgery), glaucoma subtype, follow-up time and the baseline IOP. As the IOP reduction expressed as percent reduction from baseline IOP is not normally distributed, ΔIOP was used as the dependent variable instead. The results are summarised in Table 2. There was no correlation of either TO opening or ACD with ΔIOP. Besides baseline IOP, all added covariates, including follow-up time and the change in number of eye drops had no influence on ΔIOP.

**Table 2:** Results of the multiple linear regression model

Multiple linear regression analysis (dependent variable: ΔIOP)			
Parameter	Estimate	95% Confidence interval of estimate	*p *value
TO opening	0.0016	−0.025/0.028	0.90
ACD	-1.90	−5.53/1.73	0.30
Lens status phakic (standard: combined surgery)	1.61	−0.37/3.60	0.11
Lens status pseudophakic (standard: combined surgery)	0.89	−1.34/3.11	0.43
Change in number of eye drops	−0.16	−0.77/0.45	0.61
Follow-up time	−0.0002	−0.013/0.013	0.98
Baseline IOP	−0.87	−9.0/0.096	**<0.0001**

**Fig. 4:**
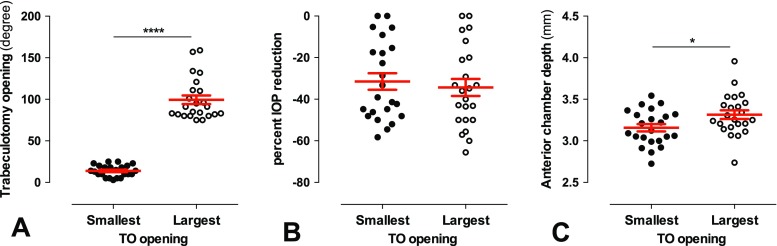
Comparison of the quartiles with the smallest and the largest TO opening (*n* = 23 for each group). A) Validation of the difference between the groups: The TO opening was significantly different (*p* < 0.0001). B) The smallest and largest TO opening group showed an almost equal mean and range of percent IOP reduction (*p* = 0.64). C) A large TO opening was associated with a significantly deeper AC (*p* = 0.041)

As testing for a linear relationship between TO opening and IOP reduction and ACD did not exhibit a significant correlation, we sub-analyzed in a more simple approach whether at least the extremes of the TO opening differed in their corresponding percent IOP reduction and ACD. Thus, the smallest and the highest TO opening quartile (*n* = 23 each group) were compared (Fig. 4). While TO opening was highly significantly different between the two groups [smallest: 13.9 ± 1.4; largest: 99.4 ± 5.2° (mean ± SEM); *p* < 0.0001, unpaired *t* test; Fig. 4a], no significant difference was detected with regard to the IOP reduction [smallest: −31.5 ± 4.0%, largest: −34.3 ± 4.1% (mean ± SEM); *p* = 0.64; Wilcoxon rank sum test; Fig. 4b]. However, the largest TO opening quartile exhibited significantly higher ACD values than the smallest quartile (smallest: 3.16 ± 0.04 mm; largest: 3.32 ± 0.05 mm; *p* = 0.031; Wilcoxon rank sum test with Bonferroni–Holm correction for multiple comparison; Fig. 4c).

### Subgroup analysis of trabeculotomy cases without cataract surgery

**Fig. 5:**
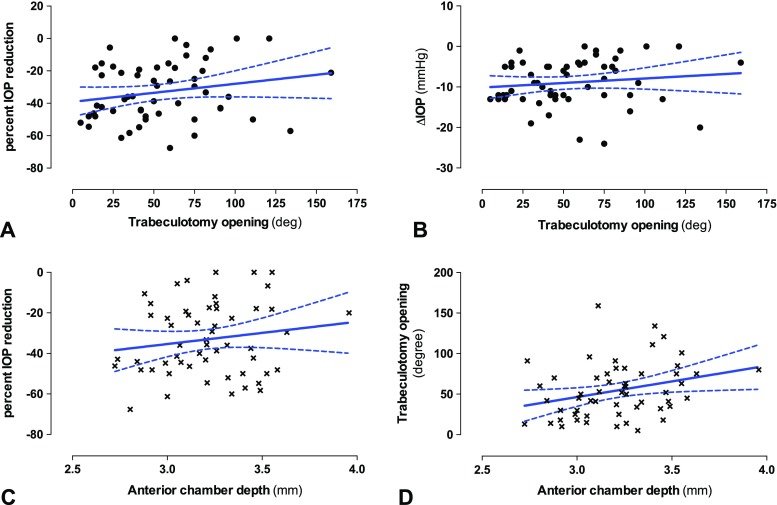
Scatterplots and linear regression analysis for the subgroup of cases where only trabeculotomy without cataract surgery was performed (*n* = 55): TO opening versus percent IOP reduction (A) or ΔIOP (B). Similar to the result of the whole study population, the slope of the linear regression line was close to zero and the 95% confidence interval of the slope included zero for both plots, indicating no correlation between TO opening and IOP reduction. C) ACD versus IOP reduction was also not significantly correlated. D) The slope of the regression line between ACD and TO opening was significantly non-zero (*p* = 0.03)

**Fig. 6:**
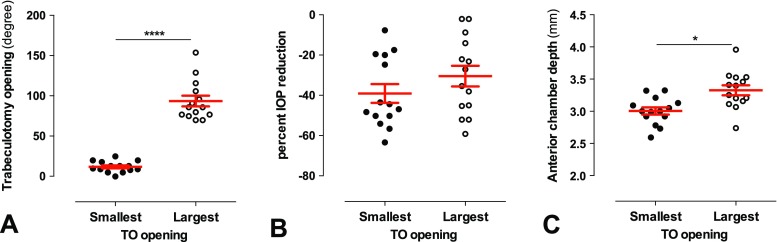
Quartile comparison (1st versus 4th TO opening quartile) for the trabeculotomy-only subgroup (*n* = 14 for each quartile). A) The TO opening was highly significantly different between both groups (*p* < 0.0001). B) Similar to the whole study population, the IOP reduction was not significantly different between the quartile groups (*p* = 0.28). C) The largest TO opening (4th) quartile showed a significantly deeper AC than the smallest TO opening (1st) quartile (*p* = 0.02)

As cataract surgery itself might have had an influence on IOP reduction as well as on ACD, we separately analyzed the subgroup of trabeculotomy cases without additional cataract surgery (*n* = 55). Compared to the whole study population, nothing changed regarding the relationship between IOP reduction and TO opening: No significant correlation was found neither in the scatter plot with linear regression analysis[(Fig. 5a (percent IOP reduction) and B (ΔIOP); slope for 5A = 0.11; 95% CI: -0.03 to 0.25; R^2^ = 0.04; slope for 5B = 0.02; 95% CI: -0.02 to 0.07; R^2^ = 0.02] nor in the comparison between the 1st and 4th quartile of TO opening (Fig. 6a and b; 1st quart. IOP reduction: −37 ± 4.7%, 4th quart. IOP reduction: −28 ± 5.1%; *p* = 0.28). Regarding ACD, no correlation was found with the amount of IOP reduction (Fig. 5c). However, in this subgroup, a deeper AC was correlated with a larger post-operative TO opening in both the linear regression analysis (Fig. 5d; slope = 38.6; 95% CI: 3.7 to 73.6; R^2^ = 0.08; *p* value for the slope being non-zero: 0.03) and in the comparison between the 1st and 4th TO opening quartile (Fig. 6c; mean ACD 3.07 ± 0.05 vs. 3.3 ± 0.08 mm, respectively; *p* = 0.02).

### Intraoperative blood reflux and IOP reduction

The reflux of blood through collector channels into the AC may indicate the patency of the aqueous outflow system distal to Schlemm’s canal. In our study, the surgeon rated the amount of intraoperative blood reflux to no, mild, moderate or strong. When the resulting IOP reduction of these blood reflux subgroups was compared, no significant difference of their mean IOP reduction was seen (*p* = 0.93, ANOVA; data plot in supplemental Fig. 3).

All eyes in the strong reflux blood group (*n* = 8) had an IOP reduction of more than 20%, while for the mild reflux group, it was only 27 out of 40 (67,5%) and for the moderate reflux group, 32 out of 43 (74,4%) eyes. These differences were not statistically significant (*p* = 0.16, Kruskal–Wallis test). The group size of ‘no blood reflux’ was too small (*n* = 2) to be included in the analysis.

## Discussion

The TM is the structure considered to provide the highest resistance to aqueous outflow [1, 2]. This suggests that the pressure-lowering effect of the trabeculotomy increases with the amount of tissue that was removed. A prerequisite for this hypothesis is that Schlemm’s canal is divided into sub-segments by a number of thin septae, which has been shown by histological studies [15], preventing aqueous humor from a circular flow through the channel and gaining access to the entirety of collector channels by any trabeculotomy opening regardless of its size.

In our study, we did not detect a correlation between the TO opening size and IOP reduction. A possible explanation may be that Trabectome surgery induced changes to the outflow structures distal to the TM (collector channels/aqueous veins), such as obstruction of the channel/vein lumina with debris or occlusion of collector channel ostia by mechanical trauma or post-operative inflammation. However, it is also conceivable that the distal outflow pathway was already dysfunctional before surgery. This would imply that in a significant subset of glaucoma patients, a pathological impairment of collector channel or aqueous vein function may play a major role in glaucomatous IOP elevation. Supporting this hypothesis, previous publications have shown various morphological alterations of the distal outflow pathway in glaucoma eyes (summarised in Hann et al., 2011 [10]). Moreover, a recent publication reported the intraoperative observation of fluid waves in episcleral veins, which was correlated with better post-operative IOP reduction [16]. This indicates a considerable variability of the flow capacity of the intrascleral aqueous drainage system and, secondly, that a functional intrascleral drainage system distal to the trabeculotomy site is prerequisite for good IOP reduction.

Our study population also included combined cases of trabeculotomy and cataract surgery. As the cataract surgery might have biased our data regarding the anatomy of the chamber angle as well as the ACD measurements (see also below), we built a subgroup of trabeculotomy-only cases (*n* = 55). The results were comparable to the original study population, indicating that the possible influence of the cataract surgery was not significant. This was also supported by the multiple linear regression data, which did not show a significant influence of the combined surgery on IOP reduction.

Trabectome surgery has been shown to be particularly effective in exfoliation glaucoma (XFG) [8], suggesting that the paradigm of an increased outflow resistance mainly at the TM is particularly true for this glaucoma entity. In our XFG subgroup, IOP reduction is higher than in the primary open angle glaucoma (POAG) subgroup (−42.6 ± 3.6% and −29.7 ± 2.3%, respectively; *p* = 0.0026). Interestingly, in the XFG subgroup, the linear regression line had a negative slope, indicating that a larger TO opening might be related to better IOP reduction (supplemental Fig. 2). However, the variability of the IOP reduction was relatively high, particularly in the small TO opening range, and the number of data points (*n* = 24) was too small for valid statistical testing. Larger studies focused on exfoliation glaucoma may, therefore, be worth pursuing.

The intra-observer ICC of the TO opening measurement was 0.993, which we consider sufficiently high. Thus, a significant impact on the regression analysis is unlikely. Moreover, the direct comparison of the smallest/largest TO opening quartile group should be relatively robust against a small to moderate measurement error. This quartile comparison illustrates that an equally wide spectrum of IOP reduction occurred in both conditions, very small and very large TO openings.

Unlike IOP reduction, the ACD measurements were significantly higher in large TO openings. We hypothesize that a deeper AC reduced the extent of re-closure of the initial TO opening most likely due to the larger irido-trabecular distance. However, the relevance of this finding remains unclear as in the multivariate analysis the ACD was not correlated with IOP reduction.

A limitation of our study is its retrospective nature. As opposed to a prospective study, it contains certain variability regarding the follow-up time and the extent of glaucoma medication pre- and post-operatively. For the latter point, the IOP reduction might have been a result of the surgical effect *and* of the change of glaucoma medication. We, therefore, included the change of glaucoma medication, as quantified by the number of glaucoma drugs pre- and post-operatively, as a covariate in our multiple linear regression model, which had no significant influence on the IOP reduction (*p* = 0.61). However, the number of glaucoma drugs is only a coarse approximation to the individual IOP reduction by medication and, therefore, an interference of the glaucoma medication change with the IOP reduction cannot be ruled out completely.

In conclusion, the fact that the TO opening did not determine the degree of IOP reduction in our study points to the poorly characterised role of the intrascleral outflow system in glaucoma as well as to its potential alterations by the surgical procedure itself.

## Electronic supplementary material

Supplemental Video 1 Representative sequence of radial OCT sections used to measure the TO opening. The first image is the horizontal section also used to measure the ACD. In this image, the TO opening is visible in the left (nasal) part. From the horizontal position, the scan runs first clockwise, showing the superior part of the trabeculotomy opening. Then, it jumps back to the horizontal section and starts running counterclockwise, showing the inferior part of the TO opening, which is now visible on the right side. For this dataset, Schlemm’s canal was measured open over 60 degrees. (MOV 19469 kb)

Supplemental Figure 1 **A)** Bland–Altman chart of the TO opening intra-observer variability (*n* = 15). Bias (interrupted line) was 1.7 degrees, 95% limit of agreement (dotted lines) was ±10.4 degrees. **B)** Bland–Altman chart of the ACD measured before and after cataract surgery in a separate group of 10 eyes of 10 patients to test for the possibility of a systematic error due to cataract extraction in combined surgery cases. No significant systematic change of the ACD measurement by cataract surgery alone could be detected in this sub-study. Interrupted line: bias; dotted lines: 95% limit of agreement; gray lines: linear regression line with 95% confidence interval of slope. (JPEG 31 kb)

High-resolution image (EPS 103 kb)

Supplemental Figure 2 Scatterplot and linear regression analysis between TO opening and percent IOP reduction for the XFG subgroup (*n* = 24). The 95% confidence interval of the slope included zero. (JPEG 27 kb)

High-resolution image (EPS 109 kb)

Supplemental Figure 3 Scatterplot of the percent IOP reduction for the intraoperative blood reflux subgroups. The mean IOP reduction was not significantly different between groups. When applying a −20% IOP reduction success criterion, all eyes in the strong reflux group were within this limit, while it was only 67.5% and 74.4% in the mild and moderate group, respectively. However, the differences were not statistically significant (*p* = 0.16). (JPEG 43 kb)

High-resolution image (EPS 102 kb)

## References

[1] Tamm ER (2009). The trabecular meshwork outflow pathways: structural and functional aspects. Exp Eye Res.

[2] Tektas O-Y, Lütjen-Drecoll E (2009). Structural changes of the trabecular meshwork in different kinds of glaucoma. Exp Eye Res.

[3] Francis BA, See RF, Rao NA (2006). Ab interno trabeculectomy: development of a novel device (Trabectome) and surgery for open-angle glaucoma. J Glaucoma.

[4] Minckler DS, Baerveldt G, Alfaro MR, Francis BA (2005). Clinical results with the Trabectome for treatment of open-angle glaucoma. Ophthalmology.

[5] Minckler D, Mosaed S, Dustin L, Ms BF (2008). Trabectome (trabeculectomy-internal approach): additional experience and extended follow-up. Trans Am Ophthalmol Soc.

[6] Filippopoulos T, Rhee DJ (2008). Novel surgical procedures in glaucoma: advances in penetrating glaucoma surgery. Curr Opin Ophthalmol.

[7] Wang Q, Harasymowycz P (2012). Goniopuncture in the treatment of short-term post-Trabectome intraocular pressure elevation: a retrospective case series study. J Glaucoma.

[8] Jordan JF, Wecker T, van Oterendorp C (2013). Trabectome surgery for primary and secondary open angle glaucomas. Graefes Arch Clin Exp Ophthalmol.

[9] van der Merwe EL, Kidson SH (2010). Advances in imaging the blood and aqueous vessels of the ocular limbus. Exp Eye Res.

[10] Hann CR, Bentley MD, Vercnocke A (2011). Imaging the aqueous humor outflow pathway in human eyes by three-dimensional micro-computed tomography (3D micro-CT). Exp Eye Res.

[11] R Core Team (2014). R: a language and environment for statistical computing.

[12] Wickham H (2011). The split-apply-combine strategy for data analysis. J Stat Softw.

[13] Harrell FE (2014) Dupont with contributions from C Hmisc: Harrell miscellaneous.

[14] Tukey JW (1997). Exploratory data analysis.

[15] Unger HH, Rohen J (1959). Studies on the histology of the inner wall of Schlemm’s canal. Am J Ophthalmol.

[16] Fellman RL, Feuer WJ, Grover DS (2015). Episcleral venous fluid wave correlates with Trabectome outcomes: intraoperative evaluation of the trabecular outflow pathway. Ophthalmology.

