# Supplementary figures and images for: Trabeculotomy opening size and IOP reduction after Trabectome® surgery
Source: Graefes Arch Clin Exp Ophthalmol. 2017 May 20;255(8):1643–50. doi: 10.1007/s00417-017-3683-0 (PMC5541095; doi:10.1007/s00417-017-3683-0)

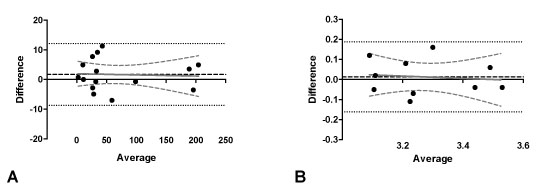

Supplement: Supplementary file 2 — A) Bland–Altman chart of the TO opening intra-observer variability (n = 15). Bias (interrupted line) was 1.7 degrees, 95% limit of agreement (dotted lines) was ±10.4 degrees. B) Bland–Altman chart of the ACD measured before and after cataract surgery in a separate group of 10 eyes of 10 patients to test for the possibility of a systematic error due to cataract extraction in combined surgery cases. No significant systematic change of the ACD measurement by cataract surgery alone could be detected in this sub-study. Interrupted line: bias; dotted lines: 95% limit of agreement; gray lines: linear regression line with 95% confidence interval of slope. (JPEG 31 kb) [file 417_2017_3683_Fig7_ESM.jpg]

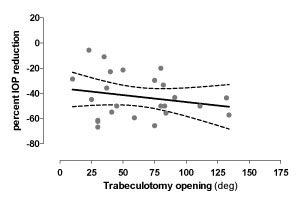

Supplement: Supplementary file 4 — Scatterplot and linear regression analysis between TO opening and percent IOP reduction for the XFG subgroup (n = 24). The 95% confidence interval of the slope included zero. (JPEG 27 kb) [file 417_2017_3683_Fig8_ESM.jpg]

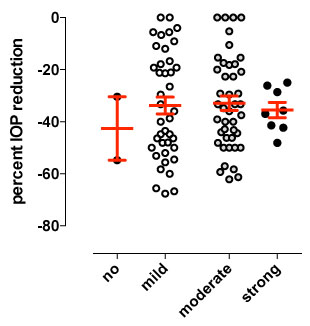

Supplement: Supplementary file 6 — Scatterplot of the percent IOP reduction for the intraoperative blood reflux subgroups. The mean IOP reduction was not significantly different between groups. When applying a −20% IOP reduction success criterion, all eyes in the strong reflux group were within this limit, while it was only 67.5% and 74.4% in the mild and moderate group, respectively. However, the differences were not statistically significant (p = 0.16). (JPEG 43 kb) [file 417_2017_3683_Fig9_ESM.jpg]
